# Cerebral Microcirculation, Perivascular Unit, and Glymphatic System: Role of Aquaporin-4 as the Gatekeeper for Water Homeostasis

**DOI:** 10.3389/fneur.2021.767470

**Published:** 2021-12-13

**Authors:** Jacek Szczygielski, Marta Kopańska, Anna Wysocka, Joachim Oertel

**Affiliations:** ^1^Department of Neurosurgery, Institute of Medical Sciences, University of Rzeszów, Rzeszów, Poland; ^2^Department of Neurosurgery, Faculty of Medicine and Saarland University Medical Center, Saarland University, Homburg, Germany; ^3^Department of Pathophysiology, Institute of Medical Sciences, University of Rzeszów, Rzeszów, Poland; ^4^Chair of Internal Medicine and Department of Internal Medicine in Nursing, Faculty of Health Sciences, Medical University of Lublin, Lublin, Poland

**Keywords:** aquaporin-4, glymphatic system, brain edema, neruovascular unit, cerebral fluid homeostasis

## Abstract

In the past, water homeostasis of the brain was understood as a certain quantitative equilibrium of water content between intravascular, interstitial, and intracellular spaces governed mostly by hydrostatic effects i.e., strictly by physical laws. The recent achievements in molecular bioscience have led to substantial changes in this regard. Some new concepts elaborate the idea that all compartments involved in cerebral fluid homeostasis create a functional continuum with an active and precise regulation of fluid exchange between them rather than only serving as separate fluid receptacles with mere passive diffusion mechanisms, based on hydrostatic pressure. According to these concepts, aquaporin-4 (AQP4) plays the central role in cerebral fluid homeostasis, acting as a water channel protein. The AQP4 not only enables water permeability through the blood-brain barrier but also regulates water exchange between perivascular spaces and the rest of the glymphatic system, described as pan-cerebral fluid pathway interlacing macroscopic cerebrospinal fluid (CSF) spaces with the interstitial fluid of brain tissue. With regards to this, AQP4 makes water shift strongly dependent on active processes including changes in cerebral microcirculation and autoregulation of brain vessels capacity. In this paper, the role of the AQP4 as the gatekeeper, regulating the water exchange between intracellular space, glymphatic system (including the so-called neurovascular units), and intravascular compartment is reviewed. In addition, the new concepts of brain edema as a misbalance in water homeostasis are critically appraised based on the newly described role of AQP4 for fluid permeation. Finally, the relevance of these hypotheses for clinical conditions (including brain trauma and stroke) and for both new and old therapy concepts are analyzed.

## 1. Introduction

Apart from the exchange of information, one of the most challenging tasks of the mammalian brain is to maintain the internal water and electrolyte homeostasis independent from the caprices of the external environment, in order to provide the neurons with nourishing substances and guarantee them a constancy of electrolyte concentration and osmolarity, required for their proper function (1, 2). As the modern techniques of histopathological and physiological research developed, the various tasks regarding global cerebral function have been attributed to the different cellular and acellular components of the brain tissue. Here, the neurons as the cells generating and propagating electrical impulses (which is considered as the major task of the whole brain), have been accorded the exclusive role of managing the information. Meanwhile, other brain components only play a minor role in maintaining the intracellular and molecular environment in optimal conditions for the appropriate function of the fastidious neural cells (3, 4). For instance, according to the common perception, the extracellular compartment is merely a vast space filled with a quite homogenous fluid, consisting mostly of water, substrate molecules, and the products of both the neuronal and glial metabolism floating together with nourishing vessels (2).

Certainly, this oversimplification is far from even approximating the whole complexity of the structure of brain fluid spaces, not to mention its extremely composed function regarding cerebral water turnover. The multidisciplinary research of recent years has delivered solid evidence that the intracerebral water balance is a highly complex, actively regulated process, involving all types of glia cells as well as the neurons and being highly responsible for the electrolyte and water homeostasis of the latter, thus impacting significantly the proper function of the whole central nervous system as a physiological unit (2, 4–6).

Due to a variable number of (sometimes concurring) theories, it is impossible to outline all neurobiological concepts describing how the brain water homeostasis is maintained in the limited text volume of the journal paper. Thus, the main goal of this narrative review is to provide the Reader with the critical appraisal of some of the latest ideas, which attempt to unify the recent findings in (micro-)anatomy, molecular neurophysiology, and biophysics into the form of a concise model of brain fluid turnover. In particular, the concept of the glymphatic system, conjoining the anatomic spaces filled with cerebrospinal fluid and the ultrastructures of extracellular space needs to be outlined (2, 7–10). The common denominator of all these theories is the function of cellular membrane components, called water channel proteins. Among these, particular attention was paid recently to the structure and function of aquaporins (AQPs), where aquaporine-4 (AQP4) has been acknowledged as the water channel protein of main importance for water turnover in the mammalian brain (11). First, recognized as a passive water channel, due to results of numerous neuromolecular studies, AQP4 has recently been acknowledged as an active and precise water homeostasis regulator, playing a crucial role both in physiological conditions as well as in situations where the exchange of fluids between all cerebral compartments is essential for the course of the disease (12–15). Here, the prime example is the development and subsidence of brain edema, being the major manifestation of the secondary cerebral damage in traumatic brain injury and in cerebral ischemia (16–18). For this reason, the potential of AQP4 as the target point for therapeutic methods will also be discussed.

## 2. Concepts of Cerebral Integrated Water Space

With the advent of modern neurosurgery, several concepts of cerebral fluid circulation and water turnover have been developed with the classic model of cerebrospinal fluid (CSF) flow also termed “third circulation” published by Cushing, which has since then become universally accepted (2, 19). According to his view, the brain was enveloped by the CSF layer being in constant flow. The CSF is produced in the lateral ventricles/choroid plexi, transported to the third ventricle, passing through the aqueduct and fourth ventricle, flowing to basal cisterns and distributed upon both hemispheres, where a paramedial area (superior sagittal sinus and arachnoid granulations) plays a major role in CSF reabsorption (2, 20). Already an important remark has been made, that the brain, despite its high water content lacks a usual lymphatic apparatus and lymph flow, and the CSF circulation was assumed to fulfill the role of the lymphatic circulation (provision and cleavage of water-soluble metabolites) in the brain (7, 21, 22). This macroscopic and very gross description of CSF turnover has been modified recently. In particular, the view that CSF production and resorption are the main forces behind brain fluid transportation needed to be revised (23–30). Here, the importance of perivascular spaces, called Virchow-Robin spaces (VRS) should be outlined. These fluid-filled areas, surrounding both arteries and veins running in the direct proximity or through the nervous tissue was attributed the role of the intermediate zone, joining the macroscopical subpial space, filled with CSF with the microscopically delineated extracellular area, in which single brain cells, including neurons and glia, were sustained (12, 31). Of note, in several studies, it was demonstrated that the fluid contained in VRS is moved not by simple diffusion or only due to a high pressure gradient, but is rather propelled by the pulsatile activity of arterial vessels (32–36). Such a pump mechanism seems to depend upon the cerebral microcirculation (37, 38) and the condition of disturbed vascular autoregulation impairs also the mechanism of bulk flow along the VRS (39–45). On the other hand, the raise of cerebral blood flow on the level of microcirculation can increase the dynamics of perivascular fluid (37). Clearly, cerebral microcirculation in physiological conditions relies on the metabolic demand of the nervous tissues, supplied by both blood and cerebral fluid flow (46–48). In respect to complex interactions between the cerebral vessels (including cerebral vasculature i.e., endothelial cells and pericytes, as well as astrocytes and neurons with their processes), the term neurovascular unit (NVU) has been coined. The concept of an NVU [exhaustively reviewed in (49)] encompasses these varieties of cells and their function, the interactions of which maintain the ionic, metabolic, and molecular homeostasis of the brain. In particular, the neuronal and astrocytic activity is able to provoke a dilation or a contraction of the arterial vessels [executed by smooth muscle cells (50)] or capillaries [provided by pericytes, being an integral part of NVU (51)] via a number of mediator substances, the release of which is strictly dependent on neuronal or astrocytic activity. This list includes not only the nitric oxide (NO), as the prime example of vasoactive substance (52, 53), but also products of cyclooxygenase-2 activity (prostanoids) (54, 55), D-serine of astrocytic origin (56), peptide-based vasoactive mediators including vasopressin (57), somatostatin (58), neuropeptide Y (NPY) (59, 60), and vasoactive intestinal peptide (VIP) (61), all of which the neurons or astrocytes are capable of secreting. This means, that depending on the current activity of the neurons, the autoregulation of the cerebral blood flow (on the level of microcirculation/NVU) would be able to adapt not only the blood supply but also, indirectly the control of CSF and the extracellular fluid extravasal flow (62).

Though the view of arteries and arterioles and their pulsatile action as the main pumping mechanism for cerebral fluid movement is quite straightforward and easy to understand, several physiological observations undermine this simplified concept of brain fluid mechanics (63). Here, the oscillating or even retrograde flow along VRS has been postulated and documented in several *in vivo* experiments (64, 65), drawing a conclusion that additional mechanisms exist (possibly on the molecular level) which contribute to the production, mixing, and flow of the fluid on the level of cerebral extracellular spaces. One of the most important factors is the temporal change in permeability for water and electrolytes or even larger particles of the blood-brain barrier (BBB) (66–69). The BBB, with its key component—tight junctions between endothelial cells lining the interior wall of cerebral microcirculation, used to be perceived as a seal, which prevented larger molecules from passing between the intravascular lumen and extracellular space. In this early concept, water and electrolytes were allowed to pass the BBB depending mostly on physical and chemical laws of osmosis and hydrostatic pressure (70, 71). However, the idea of BBB as the passive membrane exposed to the tides of CSF and blood circulation has been revised recently. Here, the exchange of electrolytes and larger particles (e.g., aminoacids) across the BBB has been described as an active, closely regulated process (72, 73), dependent on the energetic state of neurons, astrocytes, and endothelial cells (74, 75). The key argument, that BBB is not a passive, but an active structure, regulating the circulation of cerebral fluid on the ultramicroscopic level, was the capability of BBB to precisely regulate the amount of water passing across it. Moreover, in relation to water permeability, the BBB demonstrated high dynamics in changes of this property, both temporal and spatial (76–79). Thus, due to the rapid and physiological changes in BBB permeability to water and electrolytes, the brain can create compartments of fluid spaces, slightly but significantly different from the rest of global fluid space (80–83), in order to create a biochemical environment that is optimally adjusted to the current needs of the population of brain cells, both neurons and glia. Certainly, this dynamic function requires the presence of multiple molecular control systems, responsible for rapid changes in the transmission rate across BBB for different compounds (84). Regarding water permeability, the major control system is composed of several membrane proteins, labeled water channel proteins, with the AQP4 being appreciated as the most relevant for cerebral water turnover (85). The physiological function of AQP4 clearly results from its biochemical structure and gene expression as is described in the following chapter.

## 3. Structure, Genetics, and Distribution of AQP4

The AQP4 protein is a member of the large family of AQPs, the membrane water channels, which are widespread in all investigated organisms from bacteria and plants to vertebrates and responsible for bidirectional water permeability of phospholipid bilayers of cells (86). The AQP4 was first identified as 32-kDa mercurial-insensitive water channel in a rat lung (87) and then described in many different epithelial cells such as renal principal cells of collecting ducts, retina, iris, ciliary body, stomach parietal cells, colon epithelial cells, excretory tubules of lacrimal and salivary glands, organ of Corti, and in skeletal muscles. But it is mostly present in the mammalian brain and spinal cord, where it is localized in astrocytes directly in contact with capillaries and pia and in subpopulations of ependymal cells (88–91).

### 3.1. AQP4 Protein Structure

The structure of the monomeric subunit of AQP4 is similar for all AQPs and was at first described for AQP1 in human erythrocytes membrane (92). Any single subunit comprises two repeated segments, each built from three domains of the alpha-helix structure. All six domains (in pairs of the three) are arranged in the form of a non-polar bilayer and connected by five loops (A to E). The loops B and E (which connect the second and third domain in each segment) consist of highly conserved located motifs of three amino acids: asparagine—proline—arginine (NPA). According to the hourglass model, they cover the space between the bilayer leaflets and allow the water pore formation (92–94). The hemipore (as are also called B and E loops) is maintained by the van der Waals forces (95). The width of the pore along its lumen is not identical. The narrowest part, localized about 8Å above the center of the membrane, has a diameter of 2.8Å, similar to a single particle of water. In this site, NPA motifs make contact with each other. The pore diameter increases in the direction of the extra and intracellular layer of the membrane, which creates the hourglass-like shape of the whole structure (96). Several isoforms of AQP4 have been identified. In the rat brain, Jung et al. described two overlapping polypeptides of 323 or 301 amino acids, currently known as classical forms M1 and M23, transcribed from this same gene, but from differently localized initiation sites at the upstream (M1) and downstream (M23) of the gene. Authors have determined a polypeptide structure, similar to earlier identified AQP1, consisting of six membrane bilayer-spanning domains and five connecting loops, including hydrophobic loops B and E and containing, respectively, NPA 97–99 and NPA 213–216 sequences. The cytoplasmic amino terminus comprises both potential initiation sites, the carboxyl terminus, also localized in the cytoplasm consists of approximately 70 amino acids. Opposite to other AQPs, in the AQP4 amino acid chain no cysteine at site G94 nor at site A210, both responsible for mercurial inhibition, was found. In the amino acid sequence also three potential N-glycosylation sites were identified with the first (N153) localized in extracellular loop C. Both protein isoforms were synthesized in the presence of microsomes. When cRNA contained the downstream site, a single polypeptide of 301 amino acids and 30 kDA arose. In the presence of both initiation sites, besides the minor product, also the 323 amino acids polypeptide of 32 kDa were synthesized (93). Together with these first two AQP4 isoforms identified in humans, rats, and mice (87, 93, 97), nine AQP4 isoforms are as yet found (AQPa–f, 4, a ex and c ex) (98–100). When, as a result of the AQP4 rat gene mapping, four additional forms of AQP4 were described, the new terminology was implemented. M1 and M23 isoforms, respectively, have received names AQP4a and AQP4c, and AQP4 isoforms newly identified in rats were named AQPb and e-f (101). AQP4a, AQP4c, and AQP4e, considered classic, have six bilayer-spanning domains (1–6) and five interconnecting loops (A–E). AQP4b, AQP4d, AQP4f isoforms are devoid of helices 4 and 5 as well as connecting loop D. AQP4Δ found in human skeletal muscles is devoid of the terminal part of helix 5 and loop E (94). The recently identified isoforms of AQP4 in humans named a ex and c ex are characterized by C—terminal extension containing 29 amino acids (102). The AQP4 monomers independently of the isoform are organized into more complex structures in the form of tetramers, which additionally aggregate into orthogonal arrays of particles (OAPs) considerably various in respect of the size and shape as well as the isoform content (100, 103). The size of OAPs diameter evaluated by different microscopic methods reaches 100–500 nm (100) and the molecular weight of these higher-order structures is about 1,000 kDa (104). AQP4 a and AQP4 c are both incorporated into OAPs (105) as well as their extended variants AQPa ex and AQP4c ex (102). Additionally, it was reported that AQP4 a is able to attach to OAPs only in the presence of AQP4c, being the component of the OAPs core (106) and AQP4 c ex by the limitation of incorporated tetramers affect the size of OAPs (102). The AQP4e undergoes the incorporation into OAPs, while AQPs b and d do not (although both indirectly modulate the OAPs amount) and AQP4 f was not yet evaluated (100, 101, 103). Similarly, AQP4Δ lacks the ability to be attached to OAPs, but in the endoplasmatic reticulum, it exerts an effect limiting both the abundance and size of OAPs. This dominant-negative modulation is imposed through the interactions between AQP4 isoforms of the plasma membrane (99, 100).

### 3.2. AQP4 Gene Arrangement

All AQP4 isoforms are coded by a single copy of the gene localized in humans on chromosome 18 at the junction of q11.2 and q 12.1 (97, 98). As with other AQPs, the gene coding AQP4 consists of four exons including, respectively, 127, 55, 27, and 92 amino acids, between which three introns of 0.8, 0.3, and 5.2 kb are located. The unique feature, distinguishing the *AQP4* gene from other *AQPs* genes is an alternative initiation sequence situated 2.7 kb upstream and named exon 0. It allows, after the splicing process, to encode the M1 and next 10 amino acids by exon 0 and subsequent 11 amino acids with M23 by exon 1 (97). In the promoter region, such regulatory elements as TATAAAA (TATA box) at 385 bp upstream from initiation codon, one CAAT box, and AP-1 were identified and additionally SP1, two E-boxes, two AP-2, and acute phase response elements (APRE). It was shown that the transcription initiation site is located at 46 bp downstream from the TATA box. In addition, it was revealed that at 138 bp downstream of the stop codon a sequence AATAAA is situated which is the signal of polyadenylation (107). The mRNA of AQP4 b, d, and f is formed after alternative splicing omitting exon 2 from AQP4 a, c, and e, respectively (101, 107). The AQP4Δ mRNA is alternatively spliced from AQP4 a with a lack of exon 4 (99). The variants AQP4 a and c ex are extended through translational readthrough (102). In the *AQP4* gene, numerous polymorphic sites were reported across the entire gene including coding and non-coding regions, as well as 3' and 5', flanking regions (108), but the gene is considered as highly conservative and non-synonymous single nucleotide polymorphisms (nsSNPs) are rather rare (approximately 1–2% allele frequencies) (109). Several known nsSNPs influence the protein structure and function. The occurrence of variants I128T, D184E, I205L, M224T, and M278T, although all are localized relatively far from the NPA motifs, affect protein stability. The Ile-Thr substitution in position 128 results in the change of hydrophobic to hydrophilic residue in the transmembrane region and Met-Thr substitution exerts a similar effect in a loop if it involves position 224 or the C—terminal domain and position 278. Additionally, the substitution Met—Thr deprives the amino–acid residue of a sulfur atom. The chemical relevance of two other substitutions Asp—Glu and Ile—Leu is less significant. Nevertheless, all five nsSNPs impact the AQP4 function—I128T, D184E, I205L, M224T reducing, and M278T increasing water permeability (109).

### 3.3. AQP4 Distribution

As it was mentioned AQP4 is found predominantly in the astrocytes, but the AQP4 gene expression is different in various areas of the central nervous system (CNS) with the highest levels detected in astrocytes localized near the subarachnoid space, along ventricles and blood vessels. Also in areas engaged with water balance maintaining and responsible for the osmoregulation such as the supraoptic nucleus or subfornical organ, the intense AQP4 expression was recorded (90). The distribution of AQP4 isoforms inside astrocytes varies depending on the individual isoform. The most accurately is determined for AQP4a and AQP4c (known also as M1 and M23), being two first described and best-investigated isoforms. Both of them as well as their extended forms (AQP4a ex and AQP4c ex) were found at the plasma membrane aggregated in OAPs with the isoform c in the core of OAP and isoform an attached to c (98, 100, 105, 110). The isoform a may also occur in the plasma membrane in the simpler form of tetramers (111). The isoform e is localized not only at the plasma membrane, but also intracellularly (100, 101). Other isoforms were detected only in the intracellular structures such as Golgi apparatus (isoforms b,d, and f) or endoplasmatic reticulum (Δ4) (99, 100). Additionally, isoforms b and d were found in lysosomes and early endosomes (100, 103).

Several studies underlined the fact, that the regulation of AQP4 activity relies more on the subcellular relocation than on the expression of its gene. Both isoforms of AQP4 can be translated from the same full-length transcript by a “leaky scanning” mechanism (112, 113). Previous evidence shows that both isoforms are relocated equally and that the surface localization of AQP4 increased without changing the level of protein expression. In a study by Salman et al. mild hypothermic treatment increased the surface localization of AQP4 in human astrocytes even in the lack of significant change in total protein expression levels. Here, AQP4 mRNA increased modestly in cultured human primary astrocytes following 4 h mild hypothermia (32°C) compared with control cells grown at 37°C but this increase in transcript did not result in a change in protein level. Nevertheless, the decrease in temperature influenced the surface localization of AQP4, creating a space for the potential use of therapeutic brain hypothermia as an antiedematous treatment (114). Furthermore, analysis of Ciappelloni et al. indicated that the deleterious effect of anti-AQP4 autoantibodies involved in neuromyelitis optica (NMO) is probably based on perturbation of AQP4 surface dynamic and distribution. This impact differed between both isoforms of AQP4. Notably, in this study, the water transporting function of single AQP4 molecules remained intact despite exposition to AQP4 antibodies. This puts the nanoscale distribution of AQP4 in the spotlight as a major pathophysiological mechanism and the target for potential therapeutic strategy (15, 115), see also Chapter 6.

## 4. Aquaporin 4: Its Physiological Function

The biochemical and molecular properties of AQP4 including its expression, assembly of subunits, and integration into organelle clearly define it as one of the membrane proteins. Indeed, the proper physiological function of AQP4 requires its polarized integration and anchoring into astrocytic cell membranes (116–119) and this process is regulated already at the stage of translation and protein folding (120). In particular, the location of the AQP4 along the parts of astrocytic membranes reflects its crucial function in regulating the water exchange between intra- and extravascular space: the density of AQP4 arrays is about 10 times higher in endfeet areas adjacent to cerebral microvasculature than in other zones (90, 117, 121) and this inhomogeneous localization seems to be crucial for the BBB integrity (122, 123). But even if the majority of AQP4 complexes are located in endfoot areas, the presence of AQP4 has also been demonstrated in astrocytic membrane zones, directly neighboring synaptic areas (124, 125), in particular excitatory synapses (90). This localization of AQP4 defines its main physiological functions: a direct impact on the clearance of water and cellular metabolites, altering extracellular fluid dynamics, and (most probably indirect and less precisely described) regulation of neuronal and synaptic activity including plasticity (thus impacting memory and behavior). Certainly, the role of AQP4 and the whole AQP family in the physiology of the nervous system is not limited to these two domains. Currently, up to 13 different AQPs have been identified. The diversity of their physiological roles comprises physiological solute transport including glycerol, ammonia, urea, carbon dioxide, and hydrogen peroxide (126). The permeability of water channels for different small, polar substrates depends not exclusively on transmembrane proteins, which form a more narrow or wider space but expresses considerably more complex interactions between the features of the solute as well as the pore constriction and polarity. Especially important in the highlighting of these phenomena seems to be recently described relevance between the single amino acid substitutions within the aromatic/arginine (ar/R) motifs known as the selectivity filters of different AQPs and between glycerol and urea permeability. In AQP4 the ar/R- motif is formed by phenylalanine in position 1, histidine, in position 2, and, being a small residue, alanine in position 3. *In vitro*, the mutagenesis of ar/R motifs of AQP4 consisting in substitution of histidine in position 2 and arginine in position 4 creates glycerol or urea permeable channels. The H201A and H206G substitutions, respectively, allow the glycerol and the urea permeable channels to form, while the R216A substitution creates the channel permeable for both substrates. Some authors hypothesized that the H201A mutation along with F77 composes a hydrophobic corner contacting with the alkyl chain of the glycerol due to van der Waals forces, while the loss of the alanine in the H201G mutation causes a disruption of this corner and accessibility of the V197 backbone carbonyl group for binding with water or solutes such as urea due to hydrogen bounds. Oppositely, analogous mutagenesis of AQP1 (R195A and H180/G) did not lead to the formation of urea or glycerol permeable channels (127). AQPs are also responsible for the trafficking of other membrane proteins and are involved in intercellular molecular interactions resulting in cell-cell adhesions. Due to their selectivity in ion transfer across the cell membrane and ability to counteract the osmotic changes, AQP has been attributed the role of cell volume/size regulators. As to the AQP4 itself, its role in cell adhesion (probably by facilitating aggregation or localization of other adhesion molecules) has been previously described (128, 129). For the exhaustive reviews on diversity in AQP family and AQP4 function see also (13, 130, 131), however for the sake of clarity and clinical context of this review we will focus on the AQP4 functions that are the most relevant for the function of the perivascular unit.

### 4.1. Role of AQP4 in Fluid Management

The information that is crucial for understanding AQP4 function for fluid homeostasis has been mostly (but not exclusively) gained through studies implementing animal lines with the genetic modification of AQP4 function. Accordingly, AQP4 knockout animals demonstrate enlarged interstitial fluid spaces (132, 133), increased brain water content (134, 135), and reduced capability to get rid of extracellular brain water excess (135, 136). These findings are highly suggestive of a regulatory role of AQP4 in water transportation across BBB between extracellular and perivascular space (137). Indeed, multiple attempts to trace the fluid movement demonstrated suppression of glymphatic flow in the absence of AQP4. Of note, this observation has been made not only in regard to exogenous, drug-like substances as mannitol (138) or dextran (137) but also applied to endogenous substances like tau (139–141), beta-amyloid (138, 140, 142, 143) or lipoproteins (144), which are involved in the pathogenesis of degenerative encephalopathies. Of note, the AQP4 role in facilitating the exchange of solute distribution and waste substance clearance is strongly dependent on adequate localization of AQP4 in the perivascular processes (145). Disturbance in the cell-level distribution of AQP4, as provoked by syntrophin-1-alpha *(Snta-1)* gene deletion (146) or seen in brains affected by aging (147), trauma (148), or ischemic damage (149) is related to impaired function of glymphatic clearance. Undoubtedly, it sheds new light on the role of the glymphatic system in the pathophysiology of diseases such as Alzheimer's disease or posttraumatic neurodegeneration.

Notably, based on the results of (150) and (151) a competitive hypothesis has emerged, assuming that an alternative, AQP4 independent system of fluid transportation exists. In both experiments, implementing alternative ways of tracer administration to the extracellular fluid space in experimental animals, the fluid/tracer transportation was not impacted by the AQP4 genetic status and thus by aquaporin function in both wildtype and AQP4-knockout animals.

However, the recent multicenter research effort, provided by five laboratories implementing independently developed transgenic animal models with impaired AQP function, clearly demonstrated, that transport of the tracers, cleared from extracellular space via perivascular fluid compartment is strongly dependent on the proper function of perivascular aquaporins (146).

In conclusion, the main and widely accepted role of AQP4 is the facilitation of fluid exchange between the extracellular space and the perivascular spaces (both being essential parts of the glymphatic system and incorporated in brain fluid circulation) as well as in the cleavage of several cerebral metabolites, crucial in pathophysiology of neurodegeneration. Importantly, even under the physiological condition, transportation of cerebral fluid does not represent a steady-state but is a very dynamic process constantly adapting to the current needs, being related to the energetic state of neurons and thus linked to autoregulation of microvasculature. Let us take a closer look at the previous evidence regarding this area.

### 4.2. AQP4 as a Potential Regulator of Glymphatic Flow

Soon after describing glymphatic system with the continuous fluid flow as its main function, the evidence about its dynamic adaptation to the current physiological status appeared. Of importance, the increased energetic demand of neurons on the one hand clearly increases cerebral blood flow on the level of microcirculation (152–155) [a phenomenon described as neurovascular coupling, for some recent reviews of molecular background, see also (156–159)], but on the other hand reduction of interstitial flow as the neuronal activity grew has been observed (160). More so, the conditions, that are clearly related to reduced neuronal activity i.e., sleep (161–163) and general anesthesia (164–167)—albeit in a dose-dependent manner (168) [reviewed also recently in (169, 170)]—have been associated with the enhanced glymphatic flow and interstitial fluid circulation.

Is the activity of AQP4 channels somehow responsible for this inversed relationship between neurovascular coupling and glymphatic flow? Indeed, the trend to the physiological flow reduction in regions of neuronal activation was reversed in AQP4 knockouts (171). AQP4 expression and polarization are also strongly dependent on circadian rhythm (162, 172), suggesting that proper AQP4 activity is required for physiological glymphatic stagnancy in periods/areas of neuronal excitation. Also, in clinical conditions, an increased volume of extracellular fluid/PVS spaces [as seen in AQP4 knockout animals (132, 133)] have been observed in subjects affected by neurodegenerative conditions with documented reduced daily cognitive activity (41). The linkage between neuronal excitation, increased blood microcirculation, and reduced glymphatic flow is not completely understood, but the properties of AQP4 allow us to hypothesize several interrelations between these physiological phenomena. One possibility is the direct impact of vasoactive substances on AQP4 function and expression. Indeed, NO was able to modulate AQP4 expression in cultured astrocytes via a cGMP-/ MAPK controlled mechanism (173, 174) as well as in the setting of animal experiments (175). Also, vasopressin an activation of its receptors seems to impact the density and function of AQP4 (176, 177) or AQP1 (178) channels. Finally, inflammatory vasoactive substances as thromboxane (179) seem to share AQP4 as the parallel lever of action triggering astrocytic swelling. However, some more direct and swifter response mechanisms of AQP4 response to increased neuronal activity do exist. Here, the participation of AQP4 channels in moderating the K^+^ exchange related to increased neuronal activity needs to be discussed [albeit some reports deny the importance of Kir4.1/AQP4 complex for the mechanism of astrocytic swelling (180), being proposed as the mechanism of the reduced glymphatic flow (181)]. The participation of AQP4 channels in potassium homeostasis is well-documented [as reviewed exhaustively in (130) and (182)] and relies mostly on providing the water flux necessary for spatial redistribution of K^+^ ions, released during the phase of neuronal activation (183). Importantly, the key role of AQP4 in managing K^+^ excess has been underlined by molecular studies in conditions directly related to neuronal hyperexcitation as spreading depolarization (184, 185) or seizures [both in experimental (186–190) and clinical (191–193) settings]. Since K^+^ surplus in extracellular fluid space is linked to the function of cerebral micro perfusion, including neurovascular coupling (194–197), it may be hypothesized, that disturbed AQP4 function underlies the pathophysiology of several conditions related to improper reactivity of small vessels, including migraine and cluster headache (198, 199) via this mechanism. More importantly, the disturbance in potassium homeostasis attributable to AQP4 misfunction seems to result in ischemic exacerbation of secondary brain damage as may be noticed in stroke (200–202), subarachnoid hemorrhage (203–207), spontaneous intracerebral hemorrhage (208, 209) or traumatic brain injury (210–213). With regard to these conditions, even stronger links between secondary injury and AQP4 function do exist, namely the development of brain edema, which is the most direct result of impaired cerebral fluid homeostasis.

## 5. Brain Edema and Role of AQP4 in Its Pathophysiology

Certainly, the role of AQP4 in the development and subsiding of brain edema in different cerebral pathologies is of paramount importance for our understanding of the (patho-) physiology of cerebral fluid circulation. According to the canonical concept, forged by Klatzo and his research group, there are two main forms of cerebral edema existing. Vasogenic edema is characterized by extracellular water accumulation due to BBB dysfunction and increased transcytosis of plasma elements, including water (145). In turn, in cytotoxic edema water excess is gathered inside the cells (both neurons and astrocytes), manifested by beading i.e., swelling of astrocytic cells and neuronal dendrites (214–218). This dichotomy has, later on, been refined by numerous works by Marmarou and associates, describing in detail energetic depletion as the major drive for cytotoxic edema development as well as radiological manifestation of both edema types (219–224). In more recent works, a third kind of brain swelling, namely ionic edema, is distinguished. This type of edema is characterized by an early influx of both water and sodium ions from the perivascular compartment into the brain parenchyma, predominantly into the astrocytic cells. Ionic edema usually precedes the impairment of tight junctions being the first phase of ischemia-related edema formation (217, 225) and is associated with brain swelling of cytotoxic character (145, 217). Until the appearance of AQP4 on the stage, the main role in the molecular performance of both ionic and vasogenic edema remained vacant. Upon discovery and description of AQP4 function in water transportation (both in physiological and pathological conditions), our view on extracellular space and, more recently, the glymphatic system for development of brain edema has evolved dramatically (226).

Initially, the results of the experiments both *in vivo* and *in vitro* seemed to be inconclusive, since AQP4 and its expression demonstrated both surge and depletion of its activity due to developing brain edema. Thus, Ke et al. reported a reduction of AQP4 expression in areas of the traumatically swollen brain (227) and a similar observation has been made by Kiening et al. (228) and Bixt et al. in a rat model of posttraumatic edema (229). On the other hand, Fukuda et al. reported a delayed but significant raise in AQP4 level, following the development of posttraumatic brain edema (230) in juvenile rats, and similar observation has been made in adult animals by Taya et al. (231) and Zhang et al. (232). These observations were hard to reconcile until AQP4 knockout animals were available. Here, consequent analysis of different forms of edema in diverse experimental paradigms revealed that in the models with predominating cytotoxic edema demonstrable in transient or persistent ischemia models, lack of AQP4 function resulted in reduced water accumulation (233–235) and/or improved outcome (236–238). One possible explanation of these findings is, that in the absence of AQP4 channels, water excess, that would be accumulated in the swelling astrocytes due to compensatory mechanism after energetic depletion, remains in extracellular space and is managed by the glymphatic system and transported by perivascular spaces, being less effective (141) although more abundant in AQP4 knockouts (233). In conditions of vasogenic edema, the AQP4 channels seem to play a beneficial role, helping in the transportation of the fluid excess from the interstitial space to the glymphatic system. This hypothesis is sound with the observation, that in animal models of predominantly vasogenic edema, as in hemorrhagic stroke (209, 239–241) brain infection (136, 242–245) or brain tumor/cold lesion model (136, 246) brain edema subsides more efficiently in the presence of properly functioning AQP4. Importantly, not only the crude amount of AQP4 units defines its impact on brain edema or spinal cord edema development. AQP4-related permeability of astrocytic membranes is strongly dependent from subcellular localization of AQP4 water channels (112, 114). Pivotal study of Kitchen et al. demonstrated, that relocation of AQP4 units is modulated mainly by calmodulin (CaM), binding directly with AQP4 domains, while this action is further enhanced by AQP4 phosphorylation, performed by protein kinase A (PKA) (15). Thus, subcellular localization of AQP4 particles seems to be even more important for brain edema formation than expression of *AQP4* genes.

The topic of AQP4 dual impact on brain edema development/resolution is the most clearly seen in neurotrauma research. Here, several traumatic brain injury (TBI) models exist, in which the dominance of cytotoxic or vasogenic edema type relies not only on the mechanism of primary injury but changes dynamically over time as the influence of AQP4 does. Several studies implementing controlled cortical impact paradigm (CCI) (227, 229, 247) (with an initial predominance of cytotoxic edema) demonstrated a decrease in AQP4 activity and expression accompanying edema development (227–229) [although Taya et al. (231) and Fukuda et al. (248) described an AQP4 concentration raise in early stages of CCI]. To the contrary, animal studies using fluid percussion injury (with predominantly vasogenic edema) (249, 250) or weight drop models (148) demonstrated a rise of AQP activity/expression. Notably, in models of more severe brain damage, the molecular effect of AQP4 activation may be counteracted by loss of the cells being AQP4 carriers, possibly making the interpretation of data even more difficult (251). The same refers to the models with mixed type of posttraumatic edema (232), demonstrable in several head injury studies conducted in AQP4 knockout animals, where the net differences in edema development were not as clear as in experiments, in which conditions of purely cytotoxic or purely vasogenic edema were analyzed (13, 252). Nevertheless, in long-term outcome analysis, it was documented that animals lacking AQP4 demonstrated better recovery regarding neuroinflammatory events and cognitive function (18). On the other hand, AQP4 deficiency was also associated with the lower threshold of posttraumatic seizures (188). Notably, in the animal model of minor head injury, where brain edema is of lesser relevance for the posttraumatic course, lack of AQP4 was demonstrated to be neuroprotective (253) (an effect similar to pathophysiological conditions with cerebral edema of cytotoxic type) (13). As was discussed above, previous studies have shown that AQP4 seems to have different functions and outcomes in different CNS disorders. Hence, the need for accurate and reproducible methods evaluating the activity of AQP4 should be underlined. These needs meet the recently developed calcein fluorescence assay. Shortly, calcein is a dye with fluorescent properties that is provided to plate adherent cells as the membrane-permeable and non-fluorescent acetoxymethyl ester (calcein-AM). Next, the calcein-AM is metabolized by intracellular enzymes to fluorescent calcein. Then, cell shrinkage is induced by using a hypertonic medium and the quenching fluorescence of calcein is continuously measured. The concentration-dependent fluorescence reflects cells volume and enables the evaluation of water transport across the plasma membrane. Obtained curves of the shrinkage of the cell allow quantifying relative and absolute water permeability (254). Of note, calcein fluorescent assay is only one of several *ex vivo* methods to assess AQP4 function. Here, the spectrum of methodology reaches from cell culture-based osmotic swelling tests over stopped-flow spectroscopy tests in e.g., liposome suspensions up to *in silico* computational assays. This variety of research methods should be critically considered, since every single assay carries its advantages and limitations, as outlined in exhaustive reviews of Verkman et al. (255) and Abi-Awan et al. (256).

## 6. Discussion: AQP4 as a Target for Therapeutical Approaches

Due to the ambiguous properties of AQP4 regarding its impact on water homeostasis in different types of edema, the results of experimental studies in which AQP4 function is blocked or enhanced need to be critically analyzed before being translated into clinical practice. Indeed, recently several compounds have been claimed to execute beneficial impact on the course of secondary brain damage, including brain edema via interference with AQP4 function and expression. Here, neuroprotective and antiedematous action of erythropoietin has been linked with the preservation of AQP4 function in trauma (257), hydrocephalus (258), and cerebral ischemia (259). Further, the neuroprotective action of several (food) antioxidants has been explained by the adjustment of AQP4 channel functions (260–264). Notably, the antiedematous effect of well-known osmolar drugs such as hypertonic saline and mannitol has been recently linked to modulation of AQP4-water channel permeability (265, 266). Finally, the idea of repurposing some of these well-known drugs like acetazolamide (267–269) or levetiracetam (270) was based on their presumed or proven effect on AQP4 channels. Even more promising is the therapeutic strategy, in which the AQP4 subcellular relocation as the main driver promoting brain or spinal cord edema is targeted. Here, the pharmacological inhibition of PKA and CaM as main regulators for AQP4 subcellular localization was efficient against spinal cord edema formation, breakdown of blood-spinal cord barrier, and improved functional outcome in a rat model of spinal cord injury (15). Since CaM inhibition was provided by trifluoperazine (TFP), a compound that is already approved as an antipsychotic drug, the perspective of swift clinical implementation of these experimental results emerges. Significantly, TFP has proven its neuroprotective and antiodematous effect also in experimental models of brain ischemia (271, 272). In the most recent study, implementing photothrombic stroke model, TFP has downregulated AQP4 expression, reduced the amount of brain edema, and improved the metabolic function (as demonstrated via increased glycogen level of astrocytes located in ischemic penumbra) (271).

**Figure 1:**
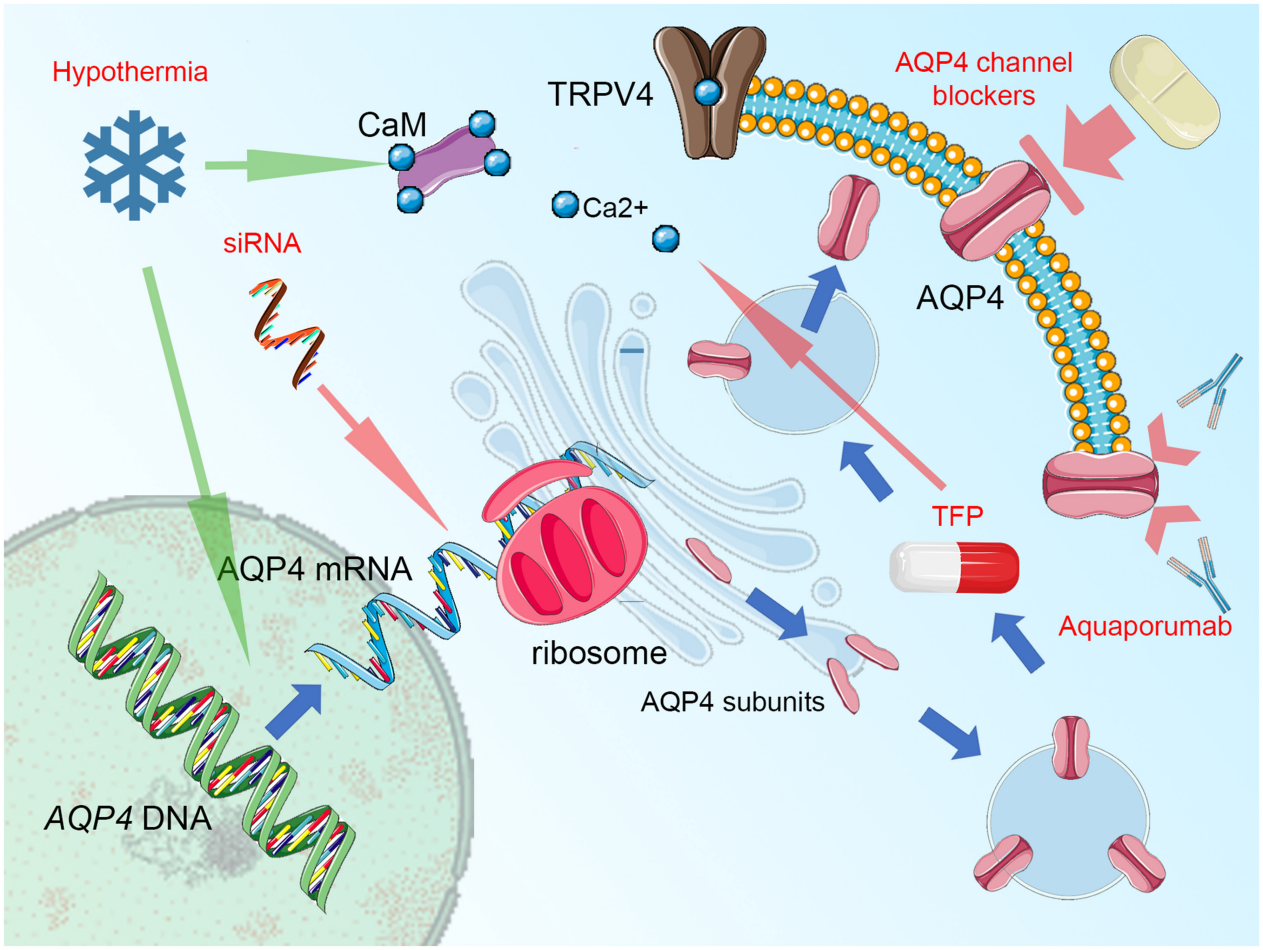
Summary figure, demonstrating aquaporin-4 (AQP4) cellular trafficking as a possible target for treatment. Blue arrows represent the process of AQP4 production and relocation, the groups of potential therapeutics are labeled by red text and their impact is marked by green (enhancing) or red arrows (blocking activity). AQP4 expression (transcription of the *AQP4* gene and translation of AQP4 mRNA with ribosomal production of AQP single subunits may be disturbed by small interfering RNA (siRNA), attaching selectively to AQP4 mRNA domains and preventing the translational readout. The single subunits of AQP4 are organized into orthogonal arrays of particles (OAPs) and as tetramers are transferred by endosomal vesicles to the proximity of cell membrane (predominantly in astrocytic endfoot area). Here, the AQP4 translocation to the cell surface takes place. This process relies on the activity of vanilloid-receptor-related subfamily 4 calcium channel (TRPV4) and calmodulin (CaM), directly binding to the AQP4 particles. Importantly, blocking CaM activity by trifluoperazine (TFP) was efficient against AQP4 relocation and the formation of cytotoxic brain edema. Notably, hypothermia exerts opposite action enhancing AQP4 surface exposition and this effect may be counteracted by TRPV4 inhibitors, Ca^2+^ chelating compounds, or CaM blockers. This effect is more relevant than the impact of hypothermia on AQP4 expression, with increased transcription reported by some, but not all relevant studies. The AQP4 channel, while integrated into astrocytic surface membrane, may be simply blocked by a number of compounds, including acetazolamide, topiramate, lamotrigine, zonisamide, acetylsulfanilamide, phenytoin, bumetanide, furosemide, tetraethylammonium, and IMD0354 as well as by heavy metal derivates or—more selectively—by TGN-020. In conditions of autoimmune response that is driven against AQP4 channels, as seen in neuromyelitis optica (NMO), blocking of antigen epitopes by monoclonal antibodies (aquaporumab), has been demonstrated as an effective NMO treatment, at least in experimental conditions. Figure created with the use of Servier Medical Art images/content of smart.servier.com in compliance with the terms of the Creative Commons Attribution 3.0 Unported Licence.

Certainly, analyses of Kitchen et al. (15) and Sylvain et al. (271) clearly document the relationship between AQP4, its subcellular location, and the beneficial role of interfering AQP4 relocalization after an injury as the main mechanism for beneficial action of TFP. Nevertheless, for most of the other studies, the question emerges: are the antiedematous or neuroprotective properties truly mediated via impact on AQP4 activity, or is the shift in AQP4 expression/function only secondary and thus reflects rather an adapting reaction of the whole glymphatic system to the beneficial action of the given drug? This question should not hinder the research community in further search for treatment strategies, in which the pivotal position of AQP4 in cerebral edema management is utilized for the improvement of outcome and neuronal protection. A good example here is the use of decompressive craniectomy. This rapid change in physical properties of the skull and brain, including hydrostatic pressure change has been associated with increased AQP4 activity, at least in areas not affected by the abundant loss of neural and glial cells (250, 251). It is imaginable, that adding AQP4-targeted therapy [like acetazolamide (267, 273) or selective AQP4 channel blocker as TGN-020, being one of the most promising candidate drugs (274–277)] to the surgical decompression would allow reducing the risk of edema surplus, related with loss of hydrostatic resistance in the decompressed brain (267). Importantly, the list of structurally non-related compounds displaying the AQP4-inhibitory properties is long and includes ethoxzolamide, topiramate, lamotrigine, zonisamide, acetylsulfanilamide, phenytoin, bumetanide, furosemide, tetraethylammonium, and IMD0354 (273, 274, 278, 279). Obviously, this list encompasses several drugs that, similar to acetazolamide, have been already approved or tested for uses other than counteracting brain edema. Hence, the strategy of drug repurposing will open a fast track for the search for efficient AQP4-targeted treatment of brain edema. The importance of this approach is underlined by the fact, that despite several assays of AQP4 water transport function are available and has been abundantly used in basic research studies [for exhaustive review see (255, 256)], no single drug exists, that has yet been approved to successfully target AQP4 water channel function in a clinical setting (256). One of the possible obstacles is the toxicity and reduced selectivity of the compounds (including heavy metal derivates), which attempted to be used according to the traditional pore-blocking approach. It is difficult to circumvent this problem, even if modern pharmacodynamic forms of drug administration (e.g., liposome-encapsulated compounds) are used (256). Unfortunately, the strategy of virtually screening myriads of candidate inhibitors does not solve this problem but rather multiplies the number of putative AQP4 blockers that fail to exert their function *in vivo*. The possible reason here is the characteristic of AQP4 molecule, with the relatively small diameter of its pore and simple structure of its molecule, that, contrary to regular membrane receptors, lacks any complex intrinsic gating and transport mechanism (255). This makes AQP4 channels less prone to be targeted by the small inhibitory molecules, dramatically shortening the list of candidate drugs (255, 256). For this reason, the use of AQP4 targeted immunotherapy or *AQP4*-gene targeted treatment should be considered. Here, in the specific condition of NMO, the anti-AQP4 monoclonal antibody (aquaporumab), competitively binding to AQP4 has proven its efficacy in reducing lesions, at least in preclinical tests (280–283). It is noteworthy to consider an antibody-based approach in conditions where AQP4 function (as cytotoxic edema, ocular neovascularization, and astroglia proliferation including glial scarring and infiltration of glial tumors) is related to exacerbation or propagation of pathologic conditions. Limiting AQP4 expression by use of small interfering RNAs (siRNA) to suppress the translation process is another viable option (256), efficiently reducing the development of posttraumatic brain edema, at least in animal models (284, 285). Finally, the implementation of physical methods interfering with AQP4 function should be mentioned. For instance, global or focal brain hypothermia seems to exert their beneficial action not only by increasing AQP4 expression (286) but also partially via impacting the function of AQP4 channels (251, 287–289). Focusing on this aspect and enhancing the impact of cerebral hypothermia treatment with AQP4-active drugs would potentially allow the second renaissance of the latter treatment mode (currently abandoned due to clinical burden of side effects, including ionic disbalance) (290, 291). The key points of cellular AQP4 trafficking that are relevant for developing new treatment strategies are outlined in Figure 1.

## 7. Conclusion

There is growing interest in the structure and function of cerebral extracellular spaces described recently as the glymphatic system. Certainly, the glymphatic flow as well as water metabolismis dependent on numerous physical laws and molecular factors. However, evidence from recent years, regarding the role of cellular water channels in physiological conditions and diverse brain pathologies clearly point out AQP4 as the key component of cerebral fluid homeostasis, acting not only as a passive channel for water and small molecular substances but playing a key role in the proper functioning of blood-brain barrier and perivascular unit. Hereby adapting the glymphatic flow to the phases of neuronal activity with increased blood flow demand in an alternating manner. The knowledge about the role of AQP4 in cerebral fluid homeostasis is vast and continually growing, however, there is still a lot to discover in this field. For this reason, as well as the ambiguity of the impact of AQP4 on the neurological outcome of cerebral edema, attempts to translate somehow the positive results of *in vivo* studies into clinical practice should await more precise and more critical benefit-risk calculations for an inhomogeneous group of conditions, in which brain edema and/or neurovascular uncoupling play a major role.

## Author Contributions

JS, MK, AW, and JO contributed the conception and design of the review. JS, MK, and AW wrote sections of the manuscript. All authors contributed to manuscript revision, read, and approved the submitted version.

## Conflict of Interest

JO is a consultant to the Karl Storz Company and receives grants from the Erbe Company. These companies were not involved in the design and workflow during the preparation of the present review. The remaining authors declare that the research was conducted in the absence of any commercial or financial relationships that could be construed as a potential conflict of interest.

## Publisher's Note

All claims expressed in this article are solely those of the authors and do not necessarily represent those of their affiliated organizations, or those of the publisher, the editors and the reviewers. Any product that may be evaluated in this article, or claim that may be made by its manufacturer, is not guaranteed or endorsed by the publisher.

## Abbreviations

AM: acetoxymethyl ester
APRE: acute phase response elements
AQP4: aquaporin-4
AQPs: aquaporins
ar/R: aromatic/arginine
BBB: blood-brain barrier
CaM: calmodulin
CBF: cerebral blood flow
CNS: central nervous system
CCI: controlled cortical impact
cGMP: cyclic guanosine monophosphate
cRNA: complementary ribonucleic acid
CSF: cerebrospinal fluid
Kir4.1: inwardly rectifying potassium channel 4.1
MAPK: mitogen-activated protein kinase
mRNA: messenger ribonucleic acid
NMO: neuromyelitis optica
NO: nitric oxide
NPA: asparagine–proline–arginine (motif)
NPY: neuropeptide Y
nsSNPs: non-synonymous single nucleotide polymorphisms
NVU: neurovascular unit
OAPs: orthogonal arrays of particles
PKA: protein kinase A
siRNA: small interfering ribonucleic acid
Snta-1: syntrophin-1-alpha
TBI: traumatic brain injury
TFP: trifluoperazine
TRPV4: transient receptor potential cation channel subfamily V member 4
VIP: vasoactive intestinal peptide
VRS: Virchow-Robin space(s).
